# The importance of early immunotherapy in patients with faciobrachial dystonic seizures

**DOI:** 10.1093/brain/awx323

**Published:** 2017-12-18

**Authors:** Julia Thompson, Mian Bi, Andrew G Murchison, Mateusz Makuch, Christian G Bien, Kon Chu, Pue Farooque, Jeffrey M Gelfand, Michael D Geschwind, Lawrence J Hirsch, Ernest Somerville, Bethan Lang, Angela Vincent, Maria I Leite, Patrick Waters, Sarosh R Irani, Faciobrachial Dystonic Seizures Study Group, Müjgan Dogan-Onugoren, Alexander Rae-Grant, Zsolt Illes, Monika Szots, Michael Malter, Guido Widman, Rainer Surges, Neil Archibald, John Reid, Callum Duncan, Anna Richardson, James Lilleker, Rafaelle Iorio, Morten Blaabjerg, Karin Abeler, Y Shin

**Affiliations:** 1 Oxford Autoimmune Neurology Group, Nuffield Department of Clinical Neurosciences, University of Oxford, Oxford, OX3 9DS, UK; 2 Dementia Research Unit, University of New South Wales, Kensington, Sydney, New South Wales, NSW 2052, Australia; 3 Krankenhaus Mara, Epilepsy Center Bethel, Bielefeld D-33617, Germany; 4 Department of Neurology, Comprehensive Epilepsy Center, Biomedical Research Institute, Seoul National University Hospital, Seoul, 110-744, South Korea; 5 Comprehensive Epilepsy Center, Department of Neurology, Yale University School of Medicine, New Haven, CT 06510, USA; 6 UCSF Department of Neurology, 675 Nelson Rising Lane, San Francisco, CA 94143, USA; 7 Comprehensive Epilepsy Service, Prince of Wales Clinical School, University of New South Wales, Sydney, New South Wales, NSW 2052, Australia

**Keywords:** faciobrachial dystonic seizures, leucine-rich glioma-inactivated 1, seizures, immunotherapy, cognitive impairment

## Abstract

Faciobrachial dystonic seizures and limbic encephalitis closely associate with antibodies to leucine-rich glioma-inactivated 1 (LGI1). Here, we describe 103 consecutive patients with faciobrachial dystonic seizures and LGI1 antibodies to understand clinical, therapeutic and serological differences between those with and without cognitive impairment, and to determine whether cessation of faciobrachial dystonic seizures can prevent cognitive impairment. The 22/103 patients without cognitive impairment typically had normal brain MRI, EEGs and serum sodium levels (*P* < 0.0001). Overall, cessation of faciobrachial dystonic seizures with antiepileptic drugs alone occurred in only 9/89 (10%) patients. By contrast, 51% showed cessation of faciobrachial dystonic seizures 30 days after addition of immunotherapy (*P* < 0.0001), with earlier cessation in cognitively normal patients (*P = *0.038). Indeed, expedited immunotherapy (*P = *0.031) and normal cognition (*P = *0.0014) also predicted reduced disability at 24 months. Furthermore, of 80 patients with faciobrachial dystonic seizures as their initial feature, 56% developed cognitive impairment after 90 days of active faciobrachial dystonic seizures. Whereas only one patient developed cognitive impairment after cessation of faciobrachial dystonic seizures (*P* < 0.0001). All patients had IgG4-LGI1 antibodies, but those with cognitive impairment had higher proportions of complement-fixing IgG1 antibodies (*P = *0.03). Both subclasses caused LGI1-ADAM22 complex internalization, a potential non-inflammatory epileptogenic mechanism. In summary, faciobrachial dystonic seizures show striking time-sensitive responses to immunotherapy, and their cessation can prevent the development of cognitive impairment.

## Teaser

Faciobrachial dystonic seizures (FBDS) are the first adult-onset autoantibody-mediated epilepsy. Thompson *et al.* describe 103 patients with FBDS, and show that seizures are responsive to immunotherapy, with early seizure cessation reducing long-term disability and preventing cognitive impairment. Potential pathogenic mechanisms include complement fixation and LGI1-ADAM22 complex internalisation.

## Introduction

Autoantibodies to leucine-rich glioma-inactivated 1 (LGI1) are associated with a limbic encephalitis. This presents with the subacute onset of frequent seizures and cognitive impairment, characterized by amnesia, confusion and executive dysfunction, often with hyponatraemia and medial temporal lobe high-signal on T_2_-weighted MRI (Thieben *et al.*, 2004; Vincent *et al.*, 2004; Irani *et al.*, 2010; Lai *et al.*, 2010). Patients improve with immunotherapies, but frequently have significant residual cognitive deficits (Butler *et al.*, 2014; Ariño *et al.*, 2016; Finke *et al.*, 2017).

In addition, LGI1 antibodies are associated with a highly specific focal seizure semiology termed faciobrachial dystonic seizures (FBDS) (Irani *et al.*, 2008, 2011, 2013; Flanagan *et al.*, 2015). Recognition of this characteristic semiology permits an accurate clinical diagnosis without ancillary testing (Sen *et al.*, 2014). Experiences from several small studies have suggested that FBDS appear preferentially responsive to immunotherapy over antiepileptic drugs (AEDs) (Irani *et al.*, 2008, 2011, 2013; Flanagan *et al.*, 2015; Navarro *et al.*, 2016; van Sonderen *et al.*, 2016). However, these studies provided limited quantification of treatment timings and minimal follow-up. Moreover, some untreated patients demonstrated improvements (Szots *et al.*, 2014). Therefore, FBDS represent an increasingly recognized condition about which the impacts of treatment type and delay have received little attention, even in the largest dedicated series of 29 patients (Irani *et al.*, 2011). Furthermore, there has been no systematic characterization of patients with FBDS without cognitive impairment. This may be especially informative as FBDS often predate the onset of cognitive impairment (Irani *et al.*, 2011, 2013; van Sonderen *et al.*, 2016). Here, we present a series of 103 patients with FBDS and LGI1 antibodies. We compare those with and without cognitive impairment, quantify the relative effects of AEDs and immunotherapy, address the hypothesis that early immunotherapy administration may prevent progression to cognitive impairment (Irani *et al.*, 2011), and explore potential pathogenic mechanisms of the LGI1 antibodies.

## Materials and methods

Standardized questionnaires (Supplementary Table 1) were distributed to an international network of clinicians who discussed patients with the Oxford Autoimmune Neurology Group between 2008 and 2013. Consecutive patients with a diagnosis of FBDS and serum LGI1 antibodies were included. Data from 103 patients described clinical and investigation features, treatment timings and modified Rankin Scale (mRS) outcomes at 12 (*n = *96), 24 (*n = *88) and 48 (*n = *55) months. Cognitive impairment was based on the clinical impression, and was well supported by available cognitive testing (Supplementary Fig. 1A). Patients and consultees underwent informed consent (approval REC16/YH/0013).

Statistical analyses were performed in R 3.2.3 and RStudio Version 0.99.491 (Supplementary material). LGI1-antibody determination with a novel flow-cytometry assay (FCA) was developed to quantify serum IgG-subclass deposition on live HEK293T cells that stably-express membrane-tethered LGI1-EGFP. ADAM22 co-transfection and soluble LGI1 were used for internalization experiments (Supplementary material).

## Results

### Demographics, phenotype and tumours

**Figure 1:**
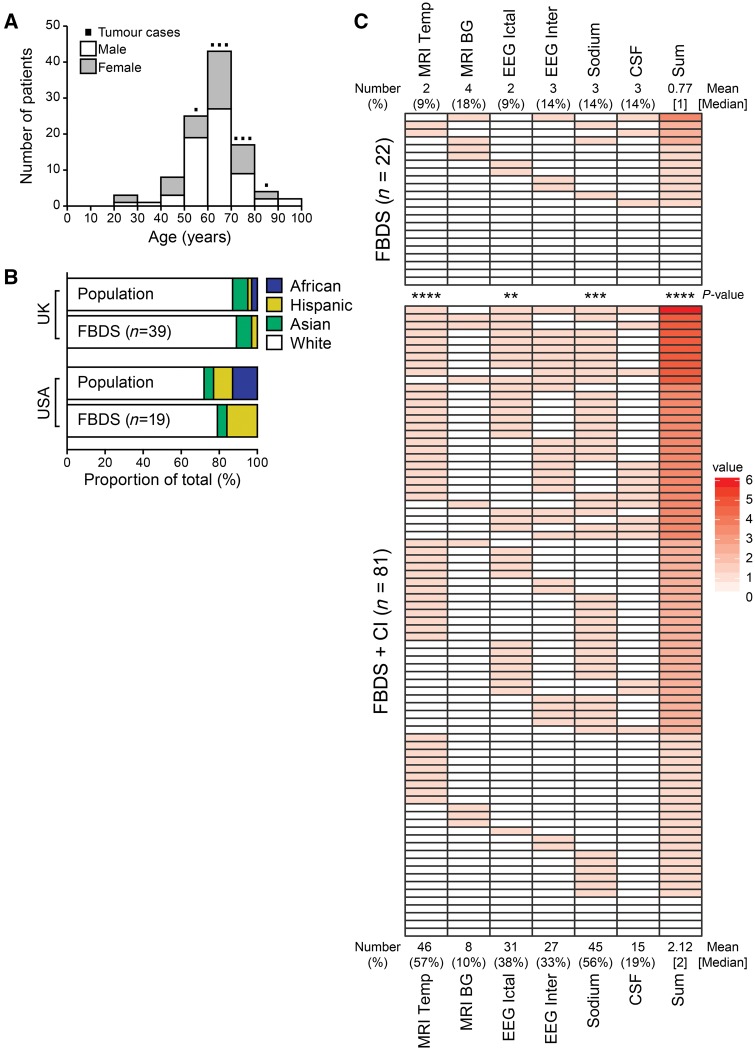
**Clinical characteristics and investigation findings in 103 patients with faciobrachial dystonic seizures.** (**A**) Age and gender distribution. The eight patients with known tumours are shown (details in Supplementary Table 2). (**B**) Ethnicity in patients with FBDS closely resembled the local population in both UK and USA, except for the absence of Afro-Caribbean cases. (**C**) Red-coloured boxes highlight abnormalities in MRI, ictal- and interictal (inter)- EEG (epileptiform features, diffuse and focal slowing), serum sodium, and CSF from patients with (*n = *81) and without (*n = *22) cognitive impairment. Sum represents the total number of abnormalities per patient. BG = basal ganglia; CI = cognitive impairment; Temp = temporal lobe. **P < 0.01, ***P < 0.001, ****P < 0.0001 (Welch’s unequal variance test for the Sum, Fisher’s exact test used for all others).

Overall, 64 of 103 (62%) patients were male, with a normal distribution centred on a median age of 64 years, and a range (22–92 years) that spared children (Fig. 1A). Only eight (8%) patients had an identified tumour, each a different type (Supplementary Table 2). Most patients with FBDS from the UK and USA were Caucasian, and no patients were of Afro-Caribbean descent (Fig. 1B). In agreement with previous smaller reports (Andrade *et al.*, 2011; Irani *et al.*, 2011, 2013; Shin *et al.*, 2013; Navarro *et al.*, 2016; van Sonderen *et al.*, 2016; Aurangzeb *et al.*, 2017), FBDS showed a high maximal frequency (median 32 per day, range 2–400), short duration (1–5 s in 74% of patients) and involvement of arm (99%), face (89%) and leg (36%) (Supplementary Fig. 1B and C). Examples are shown in Supplementary Videos 1 and 2.

### Differences between patients with and without cognitive impairment

Compared to patients identified prior to the 2011 description of FBDS, those identified after 2011 were less likely to have cognitive impairment [3/46 (7%) versus 19/57 (33%); *P = *0.013, Fisher’s], and received earlier immunotherapy (median 45 versus 112 days, *P = *0.0036, Mann-Whitney U-test). A comparison of FBDS patients with (*n = *81) and without cognitive impairment (*n* = 22; Supplementary Table 2) revealed marked similarities in demographics, frequent additional seizure semiologies and other clinical features, except hallucinations, mood and sleep disturbances, which were only observed in patients with cognitive impairment. Medial temporal lobe T_2_-hyperintensities (mostly involving the amygdala and hippocampus, *P* < 0.0001), temporal and frontal lobe ictal EEG changes (*P = *0.0096), and serum hyponatraemia (*P* < 0.0001) were almost only observed in patients with cognitive impairment (Fig. 1C). Overall, patients with cognitive impairment had more abnormal investigations than those with FBDS alone (mean 2.12 versus 0.77, *P* < 0.0001, Fig. 1C).

### Treatments administered and side effects

In total, 99 (96%) patients were administered AEDs (median = 2, range 1–10), most commonly levetiracetam (*n* = 69), sodium valproate (*n* = 37), phenytoin (PHT, *n = *32), and carbamazepine (CBZ, *n* = 26). Ninety-eight (95%) patients received immunotherapy (Supplementary Table 2): the most common immunotherapy regimes were corticosteroids alone (*n = *36; 37%), corticosteroids plus intravenous immunoglobulins (IVIG, *n = *33; 34%), corticosteroids plus plasma exchange (PLEX, *n = *12; 12%), and all three agents (*n = *12; 12%).

Strikingly, cutaneous reactions were seen in a total of 29 (29%) patients on AEDs, including five with Stevens-Johnson syndrome (Supplementary Fig. 1D). These reactions occurred across ethnicities, and were significantly associated with exposure to CBZ (48% of patients with cutaneous reactions, *P = *0.0099) and to PHT (50%, *P = *0.03; Supplementary Table 3). Side effects of immunotherapy were also common, in 43 (44%) patients, and most frequently affected mood (*n = *21) and the musculoskeletal system (*n = *11, myopathy, tendon rupture and osteoporosis). Five deaths were attributed to complications from PLEX [acute respiratory distress syndrome (*n = *2), sepsis (*n = *2) and pulmonary emboli (*n = *1)].

### Cessation of FBDS: a comparison of antiepileptic drugs and immunotherapy

**Figure 2:**
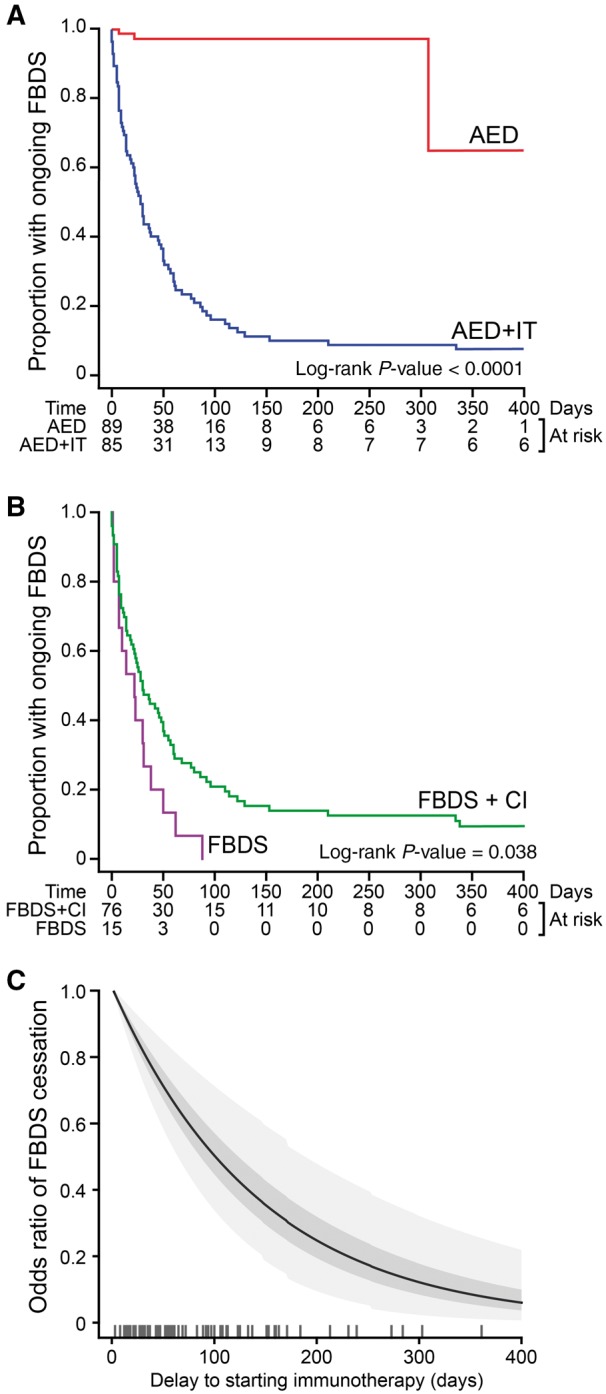
**The effects of treatments on cessation of faciobrachial dystonic seizures.** (**A**) Kaplan-Meier survival curve showing the proportion with FBDS cessation after administration of AEDs alone (red) and after subsequent additional exposure to immunotherapy (blue). (**B**) Kaplan-Meier survival curve of FBDS cessation after immunotherapy showing more rapid cessation of FBDS in patients without cognitive impairment. (**C**) Post-estimation simulation of the odds ratio of FBDS cessation against the delay to commencement of immunotherapy after the onset of FBDS. The standard error and 95% confidence intervals are shown in dark grey and light grey, respectively. Ticks represent time to starting immunotherapy. CI = cognitive impairment; IT = immunotherapy.

Given these high complication rates, the relative efficacies of AEDs and immunotherapy were investigated (Fig. 2A). Only 9 of 89 (10%) patients initially commenced on AEDs alone experienced cessation of FBDS, with a wide latency to cessation [median 186 days, interquartile range (IQR) 7–274]. These nine had a lower maximal daily frequency of FBDS (median 10 versus 40; *P = *0.0036, Mann-Whitney U-test), and no relapses [0/9 (0%) versus 30/94 (32%); *P = *0.056, Fisher’s], perhaps suggesting a milder phenotype. In contrast, in the 85 patients with subsequent additional exposure to immunotherapy, cessation of FBDS was observed in 51% after 30 days and 88% after 90 days of administration (log-rank *P* < 0.0001, Fig. 2A). Furthermore, in the three patients treated with immunotherapy alone, FBDS stopped after 2 days (*n = *2) and 14 days (*n = *1). Overall, this marked effect of immunotherapy was more pronounced in the group without cognitive impairment (*P = *0.038, Fig. 2B), and post-estimation simulations revealed that for each day immunotherapy was delayed, there was a 0.69% [95% confidence interval (CI) 0.32–1.06] relative reduction in the probability of cessation of FBDS (Fig. 2C). Consistently, multivariate regression analysis showed that time to cessation of FBDS was influenced by the number of months to treatment with immunotherapy [odds ratio (OR) 0.81, 95% CI 0.72–0.91; *P = *0.00024] and the presence of cognitive impairment (OR 0.53, CI 0.29–0.96; *P = *0.035), but not by time to AED administration, age, sex, or the frequency of the FBDS (Supplementary Table 4). In summary, whereas milder cases occasionally responded to AEDs alone, cessation of FBDS was strikingly enhanced by addition of immunotherapy, which appeared similarly efficacious in a small subset treated with immunotherapy alone.

### Factors affecting long-term disability

**Figure 3:**
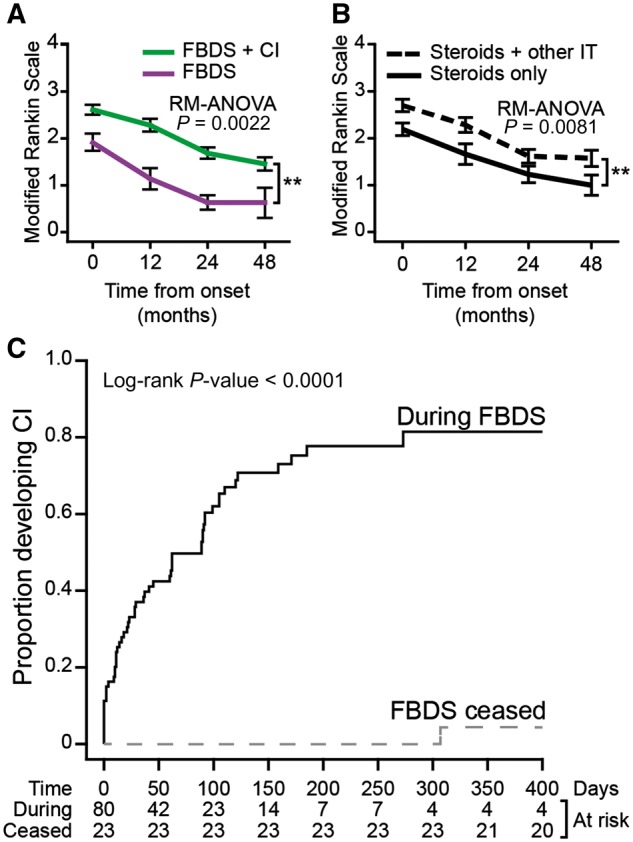
**Functional outcomes in patients with faciobrachial dystonic seizures.** (**A**) Repeat-measure ANOVA (RM-ANOVA) demonstrated a significant difference in mRS between patients with (green) and without cognitive impairment (purple). (**B**) Repeat-measure ANOVA to compare mRS between patients treated with steroids and steroids with other immunotherapy (IT) in combination (broken line), and steroids alone (solid line). (**C**) Kaplan-Meier event curve of developing cognitive impairment with ongoing FBDS (solid line), and after cessation of FBDS (broken line). CI = cognitive impairment; mRS = modified Rankin Scale.

Persistent cognitive deficits are an established feature of patients with LGI1-antibody encephalitis (Bettcher *et al.*, 2014; Butler *et al.*, 2014; Ariño *et al.*, 2016; Finke *et al.*, 2017). Overall, at disease onset, the patients without cognitive impairment showed a lower mRS (*P = *0.0032, Mann-Whitney U-test), and no cases demonstrated an inability to look after their bodily needs (mRS > 3, Supplementary Fig. 1E). Over time, the serial mRS declined at similar rates in patients with and without cognitive impairment, and poorer mRS outcomes were maintained at 4 years in patients with cognitive impairment (*P = *0.0022, Fig. 3A). Compared with patients who received corticosteroids alone, those administered additional immunotherapies had more severe disease at onset, with similar declines in mRS (*P = *0.0081; Fig. 3B). Ordinal linear regression revealed that the presence of cognitive impairment (OR 6.25, CI 2.09–20.07; *P = *0.0014) and, more modestly, the number of months to immunotherapy (OR 1.16, CI 1.02–1.34; *P = *0.031), but not time to AED administration, predicted poorer 24-month outcomes (Supplementary Table 4). Taken together, time to cessation of FBDS and longer-term outcomes were both predicted by the presence of cognitive impairment and by the time to immunotherapy administration, and additional immunotherapy did not appear to hasten recovery.

### The development of cognitive impairment with active FBDS

Next, the relative timings of FBDS and cognitive impairment were investigated. In 23 of 103 patients, FBDS were recognized after onset of cognitive impairment [median of 31 days (IQR 15–90)]. The remaining 80 (78%) patients experienced FBDS as the presenting clinical feature, and FBDS resolved with (*n = *70) or without (*n = *10) immunotherapy administration. Twenty-two of 80 never developed cognitive impairment, with cessation of FBDS after a median of 103 days (IQR 60–168). Remarkably, of the remaining 58 who developed cognitive impairment, 57 did so during active FBDS and only one did so after FBDS cessation (*P* < 0.0001, Fig. 3C). Furthermore, after 30 and 90 days of ongoing FBDS, 38% and 56% had developed cognitive impairment (Fig. 3C), respectively, suggesting a narrow therapeutic window within which FBDS cessation can eliminate the long-term disability associated with cognitive impairment.

### LGI1 antibodies: FCA, complement-fixing subclasses and LGI1 internalization

**Figure 4:**
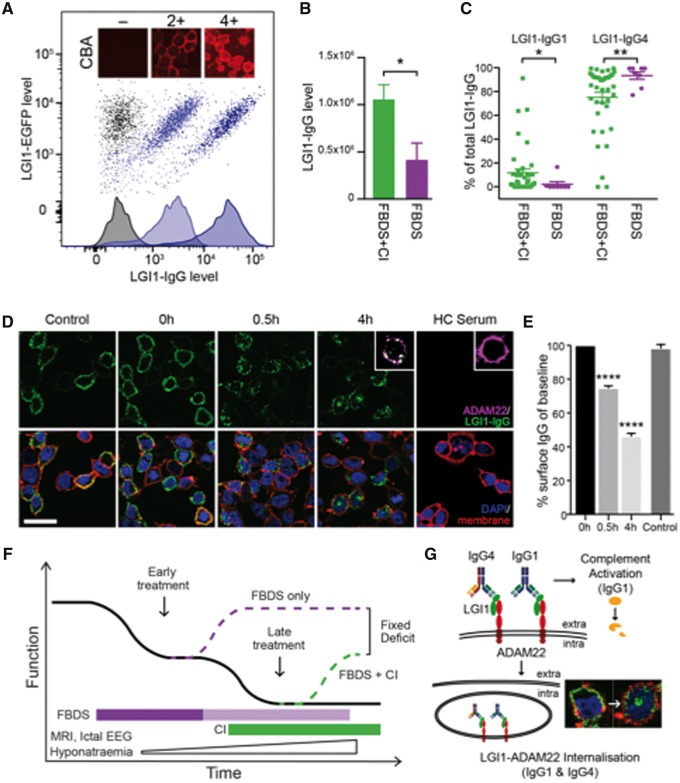
**LGI1-antibody levels, subclasses and clinical correlations.** (**A**) Flow-cytometry of stably-transfected LGI1-EGFP expressing cells labelled with IgG from a control patient (grey), and from two patients with FBDS and different LGI1 antibody levels (light blue and dark blue dot-plot clouds and histograms, median fluorescence intensities represented on both axes). (**B**) LGI1-IgG levels determined by a novel flow-cytometry assay (FCA) from 48 available initial samples are higher in patients with cognitive impairment (*P = *0.013, Mann-Whitney U-test), error bars represent standards error of the mean (SEM). (**C**) LGI1-specific IgG4 antibody and IgG1 antibody levels expressed as a percentage of the total LGI1-IgG in patients with FBDS or FBDS with cognitive impairment. *P = 0.01; **P = 0.009, both Mann-Whitney U-test. Error bars represent standard deviations (SD). (**D**) ADAM22-transfected HEK293T cells incubated with soluble LGI1 followed by sera from patients with FBDS. DAPI was used to highlight cell nuclei and PKH26 (red) to delineate plasma membranes. Scale bar = 15 µm. Surface bound IgG (at baseline, 0 h) is internalized after 0.5 h, and more so after 4 h. This was visualized from sera of patients with (*n* = 3) and without (*n* = 6) cognitive impairment, including 6/9 sera with detectable IgG4 LGI1 antibodies only. At 4°C the internalization process is inhibited and surface LGI1-IgG remains bound (control). (**E**) Quantification of **D** using flow cytometry with identically treated cells in suspension (****P < 0.0001, *n = *3 patients). (**F**) Schematic model describing effects of time from FBDS onset on clinical function including cognitive impairment, investigation results and the relative effects of early and late immunotherapy. The pathogenesis may correspond to a combination of complement deposition and LGI1-ADAM22 complex internalization, amongst other mechanisms (**G**). ADAM22 = a disintegrin and metalloproteinase domain 22; CI = cognitive impairment; LGI1-EGFP = leucine-rich, glioma inactivated 1-enhanced green fluorescent fusion protein; HC = healthy control. Experimental details provided in Supplementary material.

Next, we investigated the effects of LGI1 antibodies *in vitro*. The visual cell-based assay showed equivalent sensitivity and specificity to our novel live-cell LGI1-antibody FCA (Fig. 4A and Supplementary Fig. 2A). However, the wide dynamic range of the FCA facilitated clinical correlations in the 49 patients with available samples. The FCA detected higher overall LGI1-IgG levels in patients with cognitive impairment (*P = *0.01, Mann-Whitney U-test, Fig. 4B). As LGI1-antibody encephalitis brains show complement deposition (Bien *et al.*, 2012), LGI1-IgG subclasses were assessed. All sera contained non-complement fixing LGI1-IgG4 antibodies and 28/49 also showed complement-fixing LGI1-IgG antibodies. Patients with FBDS alone almost exclusively had LGI1-IgG4 antibodies (*P = *0.009, Mann-Whitney U-test) whereas, overall, patients with cognitive impairment showed higher proportions of LGI1-IgG1 antibodies (*P = *0.03, Mann-Whitney U-test, Fig. 4C). Furthermore, LGI1-IgG1 antibodies were undetectable in 7/8 (88%) patients without cognitive impairment versus 14/41 (34%) with cognitive impairment (*P = *0.01, Fisher’s), and greater LGI1-IgG1 proportions correlated modestly with poorer cognitive scores (*P* = 0.01 and 0.04, Supplementary Fig. 2B and C). As many of the patients recovered well with immunotherapy, we explored a potentially reversible effect of patient LGI1-IgGs in the presence of a disintegrin and metalloproteinase domain 22 (ADAM22), a known neuronal receptor for LGI1. Soluble LGI1 was transferred to ADAM22-transfected HEK cells, and incubated IgGs from patient sera were observed to internalize after 0.5 and 4 h *in vitro* at 37°C, both by visualization (Fig. 4D) and flow-cytometry quantification of surface IgG (Fig. 4E, *P* < 0.0001). Internalized LGI1-IgGs consistently co-localized with ADAM22 (Fig. 4D, inset) and internalization was observed from the sera of patients with (*n* = 3) and without (*n* = 6) cognitive impairment, and from LGI1-IgGs with both dominant IgG1 (*n* = 3) and IgG4 (*n* = 6) subclasses, but not with healthy control sera (*n* = 5) or at 4°C, a condition known to inhibit internalization.

## Discussion

This study, the largest available series of FBDS, provides a unique opportunity to describe and quantify the short- and long-term impacts of delay to treatment in this contemporary syndrome. The description of a substantial cohort without cognitive impairment has demonstrated the importance of rapid clinical recognition and prompt treatment of FBDS in the prevention of cognitive impairment. The striking effects of early immunotherapy relate to cessation of AED-refractory seizures, and the urgency of accurate diagnosis is emphasized by a quantifiable daily effect of immunotherapy delay on seizure cessation. In addition, the rapid development of cognitive impairment observed without cessation of FBDS and the effect of early immunotherapy on long-term disability, suggest prompt immunotherapy alters the clinical course of the syndrome. Furthermore, the outcomes of the groups with and without cognitive impairment did not appear to converge at 4 years, or with additional immunotherapy. This irreversible disability in patients with cognitive impairment may be secondary to their higher IgG1-LGI1 antibodies, with subsequent complement deposition and ensuing hippocampal atrophy (Bien *et al.*, 2007, 2012; Ariño *et al.*, 2016; Finke *et al.*, 2017). In contrast, the IgG4-predominant disease of FBDS alone appears largely reversible and may depend more on the non-inflammatory mechanism of LGI1-ADAM22 complex internalization. In summary, the clinical data presented allow construction of a model to describe the initiation, propagation and termination of features associated with LGI1 antibodies where early immunotherapy-based seizure cessation is key to optimizing outcomes (Fig. 4F–G).

The modal age distribution of 64 years with male predominance makes this demographic highly unusual amongst autoimmune illnesses. The absence of affected children, Afro-Caribbean patients or consistent tumour associations, all in striking contrast to patients with NMDAR-antibodies, suggests potential contributions from later life environmental triggers and genetic predispositions, such as the recently described HLA-DRB1*07:01 association (Kim *et al.*, 2017). As HLA alleles are associated with AED-related adverse effects, these may predispose to the observed frequent and often serious AED hypersensitivities.

The pathognomonic semiology of FBDS and emerging *in vitro* data (Ohkawa *et al.*, 2013; Seagar *et al.*, 2017), suggest LGI1 antibodies are epileptogenic. In these early stages of the disease, patients with an isolated seizure syndrome usually show normal investigations, suggesting a highly localized disease that is best recognized clinically. During this period, the FBDS are only minimally responsive to AEDs but exquisitely responsive to addition of immunotherapy, typically corticosteroids. The prominent dystonic posturing plus the characteristic, but not universal, involvement of the striatum (Irani *et al.*, 2011; Boesebeck *et al.*, 2013; Flanagan *et al.*, 2015) and motor cortex (Navarro *et al.*, 2016) in FBDS, suggest restricted cortical-striatal networks are initially engaged. Consistent with seizure-prone LGI1- and ADAM22-depleted animals, antibody-induced internalization of the LGI1-ADAM22 complex and related proteins may mediate this non-inflammatory epileptogenesis perhaps via disruption of molecules including AMPA receptors and potassium channels (Ohkawa *et al.*, 2013; Seagar *et al.*, 2017).

Subsequently, patients often developed cognitive impairment accompanied by an increase in both LGI1-IgG levels and frequency of FBDS (Irani *et al.*, 2011). As this study utilized clinical observations and several patients did not have demonstrable inflammation (*-itis*), cognitive impairment was preferred to the term ‘encephalitis’. This cognitive impairment became clinically apparent in over half of the patients after 3 months of active FBDS and correlated well with medial temporal lobe imaging changes, frontotemporal EEG activity and serum hyponatraemia, likely involving the LGI1-expressing, ADH-secreting hypothalamic neurons (Irani *et al.*, 2012). Therefore, cognitive impairment appeared closely associated with a transition from isolated seizures to a widespread cortical and subcortical disease with increased disability. The FBDS remained almost universally refractory to AEDs yet remarkably responsive to immunotherapy with every week of immunotherapy delay conferring a 5% relative reduction in the probability of FBDS cessation.

The remarkably rapid anti-seizure effect of early immunotherapy in FBDS, the frequent transition from FBDS alone to cognitive impairment, low frequency of IgG1-LGI1 antibodies in patients without cognitive impairment, plus previous preliminary observations (Irani *et al.*, 2011), generated the hypothesis that cognitive impairment could be prevented by termination of FBDS. This appeared to be confirmed in all but one patient, perhaps due to under-reported FBDS, subclinical seizures or a coincident neurodegenerative condition in this 70-year-old. This time-dependent intervention, alongside frequent focal seizures, raises the possibility that this syndrome may be modelled as an adult-onset epileptic encephalopathy, analogous to limbic status epilepticus (Kaplan *et al.*, 2012). However, it remains possible that FBDS and cognitive impairment represent independent manifestations of a common underlying pathology.

Early immunotherapy seems intuitive in patients with FBDS. However, several apparently intuitive medical interventions have been proven to be harmful or of no benefit (Rothwell, 2005). Therefore, moving forwards, our observations provide evidence in a disease where randomized trials may never be performed. Although the absence of prospective data collection, formal neuropsychometry or video-EEGs limited optimal assessments, they accurately reflect and inform neurology practice in this rare condition. Furthermore, the striking distinctions in investigations suggest the clinical impression of cognitive impairment relates to biological differences. While this study may not have captured patients with rapid spontaneous resolution of FBDS, the rapidity and striking effect-size upon addition of immunotherapy argue against a delayed effect of AEDs. Other study limitations include limited data regarding other seizure types, and a generic requirement for measures more tailored to these patients than the Addenbrooke’s cognitive examination-revised or mRS.

Despite the sensitivity of FBDS to immunotherapy, and the successful prevention of ensuing cognitive impairment, patients with cognitive impairment showed significant residual disability at 4 years (Bettcher *et al.*, 2014; Butler *et al.*, 2014; Irani *et al.*, 2014*a*; Ariño *et al.*, 2016; van Sonderen *et al.*, 2016). These observations suggest that although seizure recognition and termination improve long-term outcomes, an unmet medical need frequently persists. Given the observed IgG4-subclass predominance (Ariño *et al.*, 2016) suggests early rituximab may be effective but has, to date, only been trialled relatively late in the course of patients with LGI1-antibodies (Irani *et al.*, 2014*b*).

## Funding 

S.R.I. and M.M. are supported by the Wellcome Trust (104079/Z/14/Z), BMA Research Grants - 2013 Vera Down grant, Epilepsy Research UK (P1201), the Fulbright UK-US commission and by the National Institute for Health Research (NIHR) Oxford Biomedical Research Centre (BRC; The views expressed are those of the author(s) and not necessarily those of the NHS, the NIHR or the Department of Health).

## Conflicts of interest

P.W., S.R.I., A.V. and B.L. are co-applicants and receive royalties on patent application WO/2010/046716 entitled ‘Neurological Autoimmune Disorders'. The patent has been licensed to Euroimmun AG for the development of assays for LGI1 and other VGKC-complex antibodies. J.G. declares Genentech; and medicolegal consulting. Research support to UCSF came from Quest Diagnostics, MedDay and Genentech. C.G.B. gave scientific advice to Eisai (Frankfurt, Germany) and UCB (Monheim, Germany), undertook industry-funded travel with support of Eisai (Frankfurt, Germany), UCB (Monheim, Germany), Desitin (Hamburg, Germany), and Grifols (Frankfurt, Germany), obtained honoraria for speaking engagements from Eisai (Frankfurt, Germany), UCB (Monheim, Germany), Desitin (Hamburg, Germany), diamed (Köln, Germany), Fresenius Medical Care (Bad Homburg, Germany), Biogen (Ismaning, Germany) and Euroimmun (Lübeck, Germany). He received research support from diamed (Köln, Germany) and Fresenius Medical Care (Bad Homburg, Germany). He consults for the Laboratory Krone, Bad Salzuflen, Germany.

## Supplementary material

Supplementary material is available at *Brain* online.

## Appendix 1

The FBDS study working group: Dr Müjgan Dogan-Onugoren (Epilepsy Center Bethel, Germany), Dr Alexander Rae-Grant (Cleveland Clinic, USA), Prof Zsolt Illes (Department of Neurology, University of Southern Denmark), Dr Monika Szots (Department of Neurology, Mor Kaposi General Hospital, Hungary), Drs Michael Malter, Guido Widman and Rainer Surges (Epilepsy Department, University of Bonn, Germany), Dr Neil Archibald (James Cook University Hospital, UK), Drs John Reid and Callum Duncan (Aberdeen Royal Infirmary, UK), Drs Anna Richardson and James Lilleker (Salford Royal Hospital, Manchester, UK), Dr Rafaelle Iorio (Institute of Neurology, Rome, Italy), Dr Morten Blaabjerg (Copenhagen, Denmark), Dr Karin Abeler (University Hospital of North Norway) and Dr Y Shin (Seoul National University Hospital, South Korea).

## Supplementary Material

Supplementary Figure S1

Supplementary Figure S2

Supplementary Video S1

Supplementary Video S2

Supplementary Tables

## Abbreviations

AED: antiepileptic drug
FBDS: faciobrachial dystonic seizures
mRS: modified Rankin Scale
