# Supplementary figures and images for: The importance of early immunotherapy in patients with faciobrachial dystonic seizures
Source: Brain. 2017 Dec 18;141(2):348–56. doi: 10.1093/brain/awx323 (PMC5837230; doi:10.1093/brain/awx323)

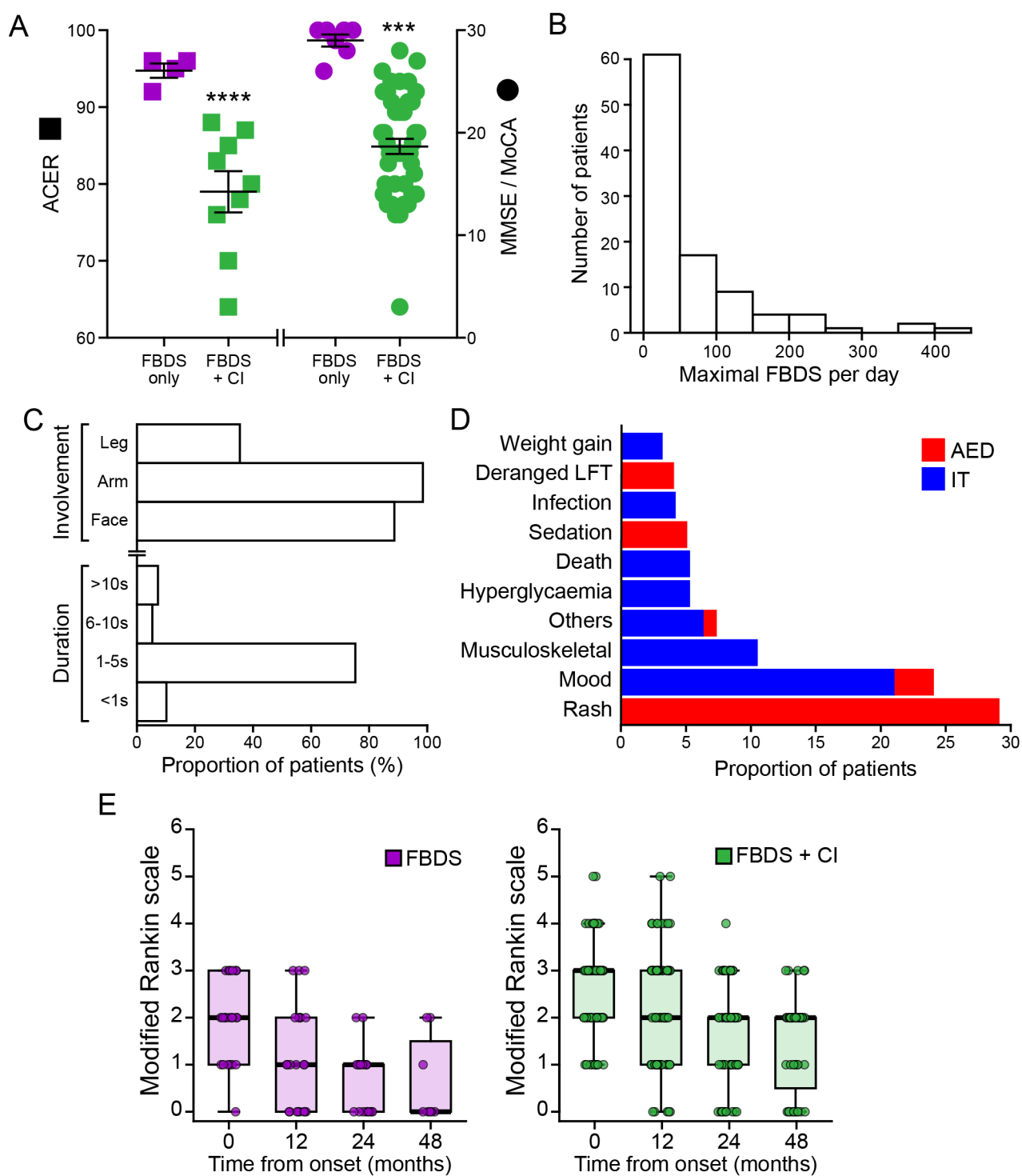

Thompson *et al.* **Supplementary Fig 1**

Supplement: Supplementary Figure S1 [file brain-2017-00524-file006_awx323.pdf]

A

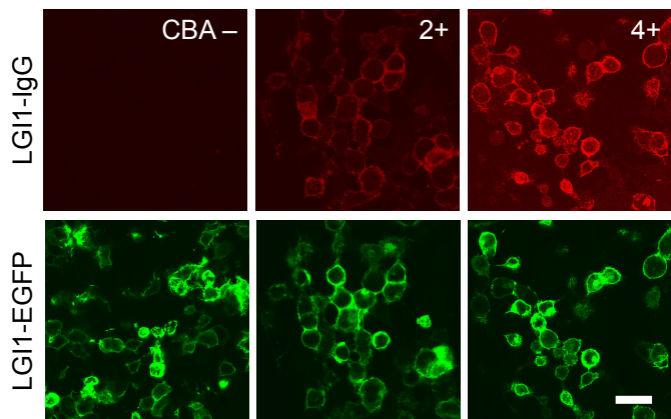

B

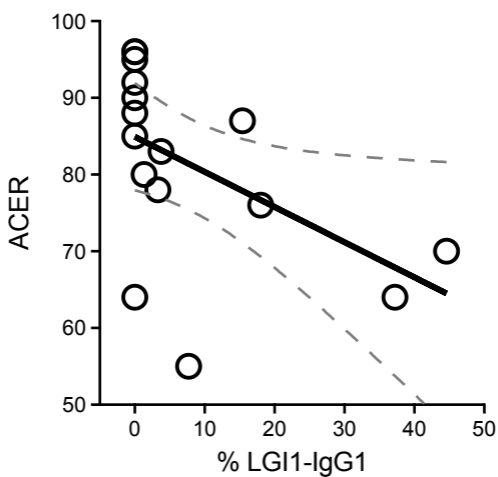

C

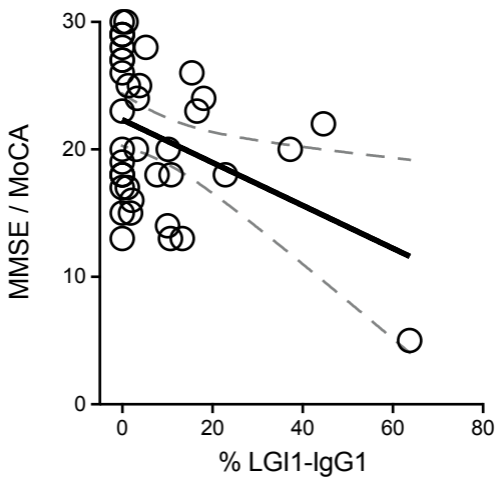

Supplement: Supplementary Figure S2 [file brain-2017-00524-file007_awx323.pdf]
