# Supplementary material for: The importance of early immunotherapy in patients with faciobrachial dystonic seizures
Source: Brain. 2017 Dec 18;141(2):348–56. doi: 10.1093/brain/awx323 (PMC5837230; doi:10.1093/brain/awx323)
Supplement: Supplementary Tables [file brain-2017-00524-file008_awx323.pdf]

Date of birth
Sex
Ethnic origin
Face (F), arm (A) and/or leg (L) affected?
Unilateral or bilateral?
If unilateral, left or right side affected?
Retained awareness during FBDS events?
Approximate length of one FBDS
Any other clinical seizures?
Video of event available?
Maximum frequency of FBDS (#per day)?
Date of FBDS onset (day/month/year)
Date of amnesia / cognitive impairment onset, if applicable (day/month/year)
Other clinical features?
Modified Rankin Scale* estimate (MRS) at symptom onset
Date anti-epileptics were 1st administered? (day/month/year)
How many anti-epileptics were administered before immunotherapy? Please list
Overall, how many antiepileptic drugs were trialled?
Were side effects attributed to antiepileptic drugs? If so, please list
What percentage of FBDS were stopped after antiepileptic drugs *alone* but prior to immunotherapy?
Date corticosteroids were 1st administered?
What approximate percentage of FBDS were stopped one month after steroids?
Total steroid dose administered?
How long were steroids continued? (total days, or date of cessation)
Were steroid-related side effects noted? If so, please list
If applicable, on which dates were other immunotherapies administered?
What percentage of FBDS were stopped one month after these other immunotherapies?
Side effects from other immunotherapies - if so, please specify?
VGKC-complex and LGI1 antibody result?
Nadir MMSE or MoCA score (/30) or Addenbrookes cognitive examination (/100), if performed
What did the MRI show when FBDS were present without cognitive impairment?
What did the MRI show when cognitive impairment was present?
Did the patient undergo prolonged EEG monitoring?
Please describe ictal EEG changes
Please describe interictal EEG changes
What were the CSF results, if performed
What was the serum sodium level when FBDS were present without cognitive impairment?
What was the serum sodium level when cognitive impairment was present?
Were any other test results abnormal? Please list
**FOLLOW UP DATA**
MRS* at latest follow up
Date of latest follow up
MMSE / MoCA / ACER score at this time is performed
Modified Rankin Scale* estimate (MRS) at symptom onset

MRS* at 12 months from onset
MRS* at 24 months from onset
MRS* at 48 months from onset
Date of FBDS cessation
Was there a relapse of FBDS?
Was the relapse after steroids were weaned?
Was a tumour detected? If so, which type?
Did the patient die? If so please provide details

* = modified Rankin Scale
0 = asymptomatic patient; 1 = symptoms do not interfere with lifestyle; 2 = symptoms lead to some restriction of lifestyle but do not prevent totally independent existence; 3 = symptoms significantly interfere with lifestyle or prevent totally independent existence; 4 = symptoms clearly prevent independent existence, although patient does not need constant attention day and night; 5 = severe disability, with patient totally dependent and requiring constant attention day and night; 6 = death due to encephalitis/FBDS

Supplementary Table 1. Questionnaire. This was electronically completed by clinicians using a right-hand column to enter answers and comments.

		All (n=103)	FBDS only (n=22)	FBDS + CI (n=81)	P value*
**Demographics**					
Age	Median (IQR)	64 (57-70)	62.5 (52-70)	64 (58-70)	0.54†
Ethnicity (%)	Caucasian	81 (79%)	21 (95%)	60 (74%)	0.14
	Asian	17 (17%)	1 (5%)	16 (20%)	
	Hispanic	5 (5%)	0	5 (6%)	
	Afro-Caribbean	0	0	0	
Tumours**		8 (8%)	1 (5%)	7(9%)	1.0
**FBDS Features**					
Regions affected (% of cases)	Face	92 (89%)	21 (95%)	71 (88%)	0.45
	Arm	102 (99%)	22 (100%)	80 (99%)	1.00
	Leg	37 (36%)	6 (27%)	31 (38%)	0.45
Side affected (% of cases)	Bilateral	45 (44%)	7 (32%)	38 (47%)	0.17
	Unilateral	58 (56%)	15(68%)	43 (53%)	
Duration (% of cases)	<1s	11 (11%)	1 (5%)	10 (12%)	0.19
	1-5s	76 (74%)	16 (73%)	60 (74%)	
	6-10s	8 (8%)	1 (5%)	7 (9%)	
	>10s	8 (8%)	4 (18%)	4 (5%)	
Frequency (/day)	Median (IQR)	32 (20-100)	35 (25-101)	31 (20-80)	0.79†
Awareness preservation (% of cases)	Yes	36 (35%)	17 (77%)	19 (23%)	<0.0001
	No or partial	67 (65%)	5 (23%)	62 (77%)	
Associated Seizure Features (% of cases)	GTCS	36 (35%)	5 (23%)	31 (38%)	0.21
	Status epilepticus	3 (3%)	0	3 (2%)	1.00
	Other focal semiologies	35 (34%)	8 (36%)	27 (33%)	1.00
Neuropsychiatric features	Hallucinations	13 (13%)	0	13 (16%)	0.065
	Sleep disturbance	19 (18%)	0	19 (23%)	0.011
	Mood	10 (10%)	0	10 (12%)	0.11
Other	Drop attacks / falls	29 (28%)	3 (13%)	26 (32%)	0.11
**Investigations**					
Hyponatremia (%)	<130	48 (47%)	3 (14%)	45 (56%)	0.00055
MRI regional abnormalities (%)	Basal ganglia	12 (12%)	4 (18%)	8 (10%)	0.27
MRI regional abnormalities (%)	Medial temporal lobe	48 (47%)	2 (9%)	46 (57%)	<0.0001
	Hippocampus	24 (23%)	1 (5%)	23 (28%)	0.021
	Amygdala	8 (8%)	0	8 (10%)	0.20

	Insular cortex	5 (5%)	0	5 (6%)	0.58
	Frontal cortex	1 (1%)	1 (5%)	1 (1%)	0.38
CSF (%)	Lymph abnormal	7 (7%)	0	7 (9%)	0.33
	Protein abnormal	13 (13%)	2 (9%)	11 (14%)	1.00
	OCB abnormal	1 (1%)	0	1 (1%)	1.00
	Any abnormal	19 (18%)	3 (14%)	16 (20%)	1.00
EEG abnormalities (%)	Abnormal Ictal EEG	30 (29%)	2 (9%)	28 (35%)	0.019
	Ictal Temporal lobe	24 (23%)	1 (5%)	23 (28%)	0.021
	Ictal Frontal lobe	10 (10%)	0 (0%)	10 (12%)	0.11
	Abnormal Interictal EEG	22 (22%)	2 (9%)	20 (33%)	0.15
	Interictal Temporal lobe	17 (17%)	2 (9%)	15(19%)	0.52
	Interictal Frontal lobe	7 (7%)	0 (0%)	7 (9%)	0.34
**Treatments**					
Antiepileptic Drugs (AED)		99 (96%)	19 (86%)	80 (98%)	0.030
AED alone		5 (5%)	2 (10%)	3 (4%)	0.29
Time to AED	Median [IQR]	30 [9-91]	23 [8-53]	30.5 [9-9[illegible]	0.49§
	Mean (SD)	67.4 (113.4)	67.5 (123.8)	67.4 (111.6)	1.00†
Immunotherapy (IT)		98 (95%)	(91%) 20	(96%)78	0.20
IT alone		4 (4%)	3 (14%)	1 (1%)	0.030
Time to Immunotherapy	Median [IQR]	61.5 [35-162]	61.5 [30-121]	92 [37-169]	0.38§
	Mean (SD)	121.7 (144.1)	121.7 (146.8)	131.1 (144.4)	0.80†
Steroids	Total	93 (90%)	19 (86%)	74 (91%)	0.44
	Alone	36 (35%)	13 (59%)	23 (28%)	0.018
	+IVIG	33 (32%)	4 (18%)	29 (12%)	
	+PLEX	12 (12%)	2 (9%)	10 (12%)	
	+IVIG & PLEX	12 (12%)	0	12 (15%)	
IVIG alone		0	1 (100%)	1.00	
PLEX alone		4 (4%)	1 (5%)	3 (4%)	1.00
Additional MMF / AZA / RTX / CP		15 (15%)	2 (9%)	13 (16%)	0.52

**Follow-up**					
mRS (median and range)	At onset	3 (0-5)	2 (0-3)	3 (1-5)	0.0032§
	12 months	2 (0-5)	2 (0-3)	2 (0-5)	0.00024 §
	24 months	1 (0-4)	1 (0-2)	2 (0-4)	0.00011 §
	48 months	2 (0-3)	0.5 (0-2)	2 (0-3)	0.038§
Relapses		32 (31%)	4 (18%)	28 (35%)	0.20

Supplementary Table 2. Demographic, clinical features, investigation results, treatments, and follow-up MRS and relapses in patients with FBDS both with and without cognitive impairment (CI). GTCS = generalised tonic-clonic seizure. *Fisher's exact test unless otherwise stated; †Welch's unequal variance t-test. §Mann Whitney U test. **Tumours included breast, bronchoalveolar, renal cell and ovarian carcinoma, parotid adenoma, renal oncocytoma, liposarcoma and liver metastases with unknown primary. CSF = cerebrospinal fluid; EEG = electroencephalogram; MRI = magnetic resonance imaging. IVIG = intravenous immunoglobulins; PLEX = plasma exchange; MMF = mycophenolate mofetil; AZA = azathioprine; RTX= rituximab; CP = cyclophosphamide.

AED	No Rash (n = 71)	Rash (n = 28)	P-value*
Carbamazepine	13 (18%)	13 (48%)	0·0099
Phenytoin	18 (25%)	14 (50%)	0·030
Barbiturates	2 (3%)	2 (7%)	0·32
Clobazam	6 (8%)	4 (14%)	0·46
Valproate	25 (35%)	12 (41%)	0·50
Oxcarbazepine	8 (11%)	5 (17%)	0·51
Lamotrigine	11 (15%)	3 (10%)	0·75
Levetiracetam	49 (69%)	20 (69%)	1·00
Lacosamide	4 (6%)	2 (7%)	1·00
Gabapentin	2 (3%)	0 (0%)	1·00
Topiramate	6 (8%)	2 (7%)	1·00
Clonazepam	11 (15%)	4 (14%)	1·00

Supplementary Table 3. Proportion of patients receiving the specific AED who developed rash. Only phenytoin and carbamazepine were significantly associated with rash. Overall, patients received between 1 and 10 AEDs each (median = 2). *Fisher's exact test. AED = antiepileptic drug.

Factors affecting time to cessation of FBDS*		
	P value	Odds ratio (95% CI)
Age	0·37	1·01 (0·99-1·03)
Sex	0·58	1·14 (0·71-1·84)
FBDS frequency	0·76	1·00 (1·00-1·00)
Time to AED	0·37	0·94 (0·83-1·07)
Time to IT	0·00024	0·81 (0·72-0·91)
Presence of CI	0·035	0·53 (0·29-0·96)
**Factors affecting mRS at 24 months****		
	P value	Odds ratio (95% CI)
Age	0·45	1·01 (0·98-1·05)
Sex	0·38	1·46 (0·63-3·42)
FBDS frequency	0·13	1·00 (1·00-1·01)
Time to AED	0·55	0·95 (0·80-1·13)
Time to IT	0·031	1·16 (1·02-1·34)
Presence of CI	0·0014	6.25 (2·09-20·07)

Supplementary Table 4. Regression analyses evaluating factors affecting time to cessation of FBDS (above) and the 24-month mRS (below). *multivariate Cox proportional hazard regression analysis; **ordinal linear regression analysis. Time to AEDs or IT presented per month. AED=anti-epileptic drugs. CI=cognitive impairment. FBDS=faciobrachial dystonic seizures. IT=immunotherapy.

Supplementary Figure 1. Features of patients with faciobrachial dystonic seizures. (A) ACER (out of 100) and MMSE/MoCA (out of 30) scores in patients with cognitive impairment (CI; green) and without CI (purple). The main cognitive deficits from ACER testing were fluency (mean 69%, range 43-86) and memory (mean 72%, range 42-92). The two patients with FBDS and CI with MMSE/MoCA>27, had ACER scores <90. (B) Distribution of maximum FBDS per day. (C) Proportion of patients with faciobrachial dystonic seizures affecting the arm, face and leg; and the duration of an individual attack (<1 second, 1-5, 6-10 or >10 seconds). (D) Side effects from treatments. Both anti-epileptic drugs (AED, red) and immunotherapies (IT, blue) were associated with frequent, but different, side effects. Musculoskeletal side effects included myopathy, tendon rupture and osteoporosis. Others included fatigue, headache (attributed to AED exposure), diarrhoea, vomiting, nausea, pain, venous thrombosis, and cutaneous bruising (attributed to IT). Death (n=5) was attributed to the complications of plasma exchange. LFT = liver function tests. (E) Boxplots of modified Rankin Scale (mRS) at 0, 12, 24 and 48 months after disease onset in patients with CI (green) and without CI (purple).

Supplementary Figure 2. Serological features and clinical correlations of patients with faciobrachial dystonic seizures. (A) Representative immunofluorescent images of cell based assay results from a healthy control serum (-) and serum from a patient with FBDS and moderate LGI1-IgG levels (2+) and FBDS with high LGI1-IgG levels (4+). Red images show patient IgG (anti-human 568 secondary antibody) and green images show distribution of LGI1-EGFP expression. Across patients, the percentage of LGI1-IgG belonging to the IgG1-subclass correlates with both the ACER score (B; r^2=0.26, p=0.04) and the MMSE or MOCA (C; r^2=0.15, p=0.01).

Supplementary methods

Statistical analyses

Fisher's exact test was used for 2x2 and higher order contingency tables; Welch's unequal variances t-test for comparison between means, and Mann-Whitney U test (MWU) for comparison of ordinal data. The survival and simPH R packages were used for Kaplan-Meier survival analysis (log-rank test p values reported), Cox proportional hazard analysis, and post-estimation simulation for hazard ratio and estimate uncertainty (Gandrud, 2015). Backward elimination regression analysis was used to build the final models whilst maintaining patient demographic variables. The car package was used for type III repeated-measures ANOVA (RM-ANOVA) testing.

LGI1-antibody determination

A novel flow-cytometry antibody (FCA) was developed to quantify serum IgG-deposition on the surface of live HEK cells engineered to stably-express a membrane-tethered LGI1-EGFP fusion protein. HEK cells were grown under geneticin selection after transfection with membrane-bound, EGFP-tagged membrane tethered LGI1 using a mammalian expression construct which has been described previously (Irani *et al.*, 2010). After growing to 80% confluency, stably-transfected cells were stained with diluted patient sera (1:50-1:800 to obtain accurate titrations) for 1 hour at four degrees Celcius. This was followed by washing in DMEM/HEPES buffer and incubation with secondary antibodies including anti-human IgG Fab (PE-labelled, Fisher Scientific), and IgG1-4 subclass-specific antibodies (Alexa Fluor 647-labeled, Southern Biotech). After further washing steps, cells were analysed on LSR Fortessa X20 (BD Biosciences). The gating strategy (using FloJo v10·0·8) excluded dead cells and doublets, and gated on cells expressing LGI1-EGFP (Fig 4A, y-axis) against anti-human IgG binding (PE, x-axis). To determine the relative quantities of total- and subclass-specific-LGI1-antibodies, a calibration curve was derived from Quantum Simply Cellular anti-Mouse IgG beads (Bangs Laboratories) coated with the same amount of secondary antibody used to stain for total- or subclass-specific-IgG on HEK cells. This assay had the same sensitivity as the previously reported visually-assessed live cell based assay (CBA, (Irani *et al.*, 2010)), and 80 sera from patients with other autoantibody-associated illnesses (40 aquaporin-4, 20 contactin-associated protein 2 and 20 N-methyl, D-aspartate antibodies) were negative on both tests.

Internalisation of LGI1-ADAM22-IgG complexes

ADAM22 cDNA was transfected into HEK 293T cells. After 48 hours of expression, ADAM22-expressing cells were incubated for 1 hour at room temperature with soluble LGI1, derived from supernatants of LGI1-transfected HEK293 cells (Irani *et al.*, 2010). Subsequently, excess LGI1 was washed away and cells were incubated with LGI1-antibody positive FBDS patient sera (30 minutes at 4°C) and secondary anti-human IgG antibody (Alexa Flour 488-labelled, 1:1000, green) for 15 minutes at 4°C. Next, cells were incubated at 37°C with a time course over 0.5 to 4 hours and stained with the membrane-label PKH26 (red, Sigma Aldrich) before fixation with 4% PFA (Fig 4D). As expected, the internalisation was inhibited at 4°C (Control). In addition, after incubation at 37°C for 4 hours with healthy control sera, strong staining was still achieved after surface LGI1-antibody application (Control). Quantification of surface IgG by flow cytometry (Fig 4E) was performed with cells in solution over the same time intervals.

Videos

FBDS Video 1. 92-year-old male with faciobrachial dystonic seizure affecting right arm and face with speech arrest. Prolonged event shown, lasting at least 11 seconds.

FBDS Video 2. 67-year-old male with a sensory premonition in his chest preceding many faciobrachial dystonic seizures. The event involves prominent dystonic posturing of the right hand and little finger in particular. The seizure is followed by manual automatisms.

References

Gandrud C. simPH: An R Package for Illustrating Estimates from Cox Proportional Hazard Models Including for Interactive and Nonlinear Effects. J Stat Soft 2015; 65: 1–20.

Irani SR, Alexander S, Waters P, Kleopa KA, Pettingill P, Zuliani L, et al. Antibodies to Kv1 potassium channel-complex proteins leucine-rich, glioma inactivated 1 protein and contactin-associated protein-2 in limbic encephalitis, Morvan's syndrome and acquired neuromyotonia. Brain 2010; 133: 2734–2748.
